# Physical Therapies for Psychosomatic Symptoms and Quality of Life Induced by Aromatase Inhibitors in Breast Cancer Patients: A Systematic Review and Meta-Analysis

**DOI:** 10.3389/fonc.2021.745280

**Published:** 2021-11-12

**Authors:** Xue-Ying Zhu, Zhong Li, Cong Chen, Ru-Li Feng, Bai-Ru Cheng, Ruo-Yi Liu, Rui-Ting Wang, Li Xu, Yue Wang, Xin Tao, Peng Zhao

**Affiliations:** ^1^ Department of Oncology, Dongzhimen Hospital, Beijing University of Chinese Medicine, Beijing, China; ^2^ Department of Cardiology, Dongzhimen Hospital, Beijing University of Chinese Medicine, Beijing, China; ^3^ Department of Encephalopathy, Dongzhimen Hospital, Beijing University of Chinese Medicine, Beijing, China; ^4^ Department of Gynecology, Dongzhimen Hospital, Beijing University of Chinese Medicine, Beijing, China; ^5^ Graduate School of Beijing University of Chinese Medicine, Beijing, China; ^6^ School of Medical Humanities, Capital Medical University, Beijing, China

**Keywords:** physical therapies, acupuncture, exercise, breast cancer, aromatase inhibitors, pain, quality of life

## Abstract

**Objective:**

To evaluate the effects of Physical Therapies (PTs) on improvement in psychosomatic symptoms and quality of life (QOL) in breast cancer patients.

**Data Sources:**

Seven databases (MEDLINE, EMBASE, Cochrane CENTRAL, China National Knowledge Infrastructure, Wangfang, VIP, and China Biology Medicine disc databases) were systematically searched from the database inception through May 18, 2021.

**Study Selection:**

Randomized controlled trials (RCTs) which compared acupuncture or exercise with a sham control or usual care for the treatment of aromatase inhibitors (AIs)-related psychosomatic symptoms and QOL.

**Data Extraction and Synthesis:**

Data were screened and extracted independently using predesigned forms. The quality of RCTs was assessed with the Cochrane Handbook for Systematic Reviews of Interventions. The effect size was calculated *via* random-effects modeling. The quality of evidence was evaluated with the Grading of Recommendations Assessment, Development and Evaluation approach.

**Main Outcomes and Measures:**

The score of pain was measured with BPI scale and Western Ontario and the McMaster Universities Index (WOMAC) scale. Emotional state was measured with Pittsburgh Sleep Quality Index (PSQI), Hospital Anxiety and Depression Scale (HADS-A), and Functional Assessment of Chronic Illness Therapy-Fatigue (FACIT-Fatigue). The QOL score was measured by self-reported measurements, including the Functional Assessment of Cancer Therapy-General (FACT-G) scale and 36-Item Short Form Survey (SF-36) scale.

**Results:**

Eleven RCTs (with 830 patients) were included in the systematic review, and data from 10 RCTs (with 798 patients) were used in the meta-analysis. Results showed acupuncture significantly reduced worst pain scores (*P* < 0.00001, *I* ^2^ = 83.5%) [SMD = −0.81, 95% CI (−1.51, −0.11)], but the effect of exercise therapies was not significant in overall change in worst pain scores (*P* =0.006, *I* ^2^ = 72.3%) [SMD = −0.30, 95% CI (−0.76, 0.16)]. Both acupuncture and exercise resulted in little to no difference in overall change in HADS-A subscale (P = 0.026<0.05, *I* ^2^ = 79.8%) [WMD = −0.21, 95% CI (−3.44, 3.03)], PSQI subscale (P = 0.488, *I* ^2^ = 0%) [WMD = 0.98, 95% CI (−0.57, 2.53)], and FACIT-Fatigue subscale (P = 0.022<0.05, *I* ^2^ = 81.0%) [WMD = 1.6, 95% CI (−5.75, 8.94)]. Exercise (compared with usual care) was associated with improving overall change in health-related QOL (subscales of SF-36 tool) (P = 0, *I* ^2^ = 72.1%) [WMD = 7.97, 95% CI (5.68, 10.25)] and cancer-specific QOL (subscales of FACT-G tool) (P = 0.304, *I* ^2^ = 16%) [WMD = 1.16, 95% CI (0.34, 1.97)].

**Conclusions and Relevance:**

This systematic review and meta-analysis suggested that based on moderate-level evidence, acupuncture was associated with significant reductions in pain intensity, and exercise might improve QOL in breast cancer patients treated with AIs. However, in psychosomatic symptoms such as anxiety, sleep disturbance, and fatigue, acupuncture and exercise training did not result in significant improvements.

## 1 Introduction

The number of breast cancer survivors is increasing as breast cancer becomes a major health concern worldwide (1). According to statistics from 185 countries in 2018, nearly 2.1 million cases of breast cancer were newly diagnosed (2). It was estimated that about 15.1% of the new cases of cancer were breast cancer, with an estimated 2.5 million new cases in China each year (3). Advances in clinical management of breast cancer increase survival rate and also cause cancer-related side effects, critically impacting on physical, psychological, and spiritual aspects of QOL (4). Endocrine therapy, especially aromatase inhibitors (AIs), is the main standard treatment for hormone-receptor-positive breast cancer (5). Eighty percent of breast cancer is hormone receptor–positive breast cancer, including progesterone receptor (PR)-positive or/and estrogen receptor (ER)-positive subtypes (6). While five years of AIs therapy can improve disease-free survival (DFS) and breast cancer specific survival (BCSS) for postmenopausal patients in an early stage of the cancer, these inhibitors are associated with several sequelae, among which is the worsening of psychosomatic symptoms (7).

AIs may induce disorders in joint and muscle, known as aromatase inhibitor-induced musculoskeletal symptoms (AIMSS), which manifest as symmetric pain or soreness in multiple joints and morning stiffness (8, 9). Psychosomatic factors play an important role in pain and physical disabilities (10). Physical symptoms such as depression can lead to chronic pain and require multiple treatment (11). Serotonin and norepinephrine influence both the progress of depression and pain because of the same neurochemical pathway. Therefore, depression and associated painful physical symptoms must be treated with equal attention (12). Illness and treatment-related distress always plagued most breast cancer patients, such as changes in body image and sexuality and fear of recurrence (13). Compared with other cancer types, breast cancer survivors showed more serious psychiatric comorbidity and psychosocial distress especially anxiety and adjustment disorders (14). In general, AIs are effective drugs for breast cancer with minimal adverse effects, including hot flashes, vaginal dryness, and headache, which are typically mild (15). However, postmenopausal breast cancer patients affected by comorbidities and treatments including estradiol reduction and cytokine dysregulation are likely to contribute to deteriorated psychosomatic symptoms, such depression and anxiety (16, 17). The abovementioned side effects strike the treatment adherence of AIs, despite the survival advantage of AIs.

In the face of the variety of psychosomatic symptoms, breast cancer patients with AIs are complex and heterogeneous. To date, no definitive pharmacological therapy has been confirmed to avoid unfavorable side effects (18). In order to meet the rehabilitation needs caused by psychosomatic symptoms, related alternative approaches have received much attention (19, 20). In recent years, physical therapy (PT) has been used to treat side effects caused by AIs (7, 21). PT is generally divided into two types: one based on using physical factors as the main means, including biofeedback and acupuncture; the other on functional training, known as the exercise therapy including yoga, aquatic exercise, tai-chi, walking, Pilates, and resistance exercises (22). Although PT, such as acupuncture, exercise, or yoga, has been shown to improve AIMSS and health-related QOL to some extent in previous published studies (18, 21, 23), the effects of PT on psychosomatic symptoms were not assessed.

Psychosomatic symptoms play a key role in the management of patients with breast cancer treated with AIs. Studies have shown that a large number of patients with AIs have poor compliance, which may impact their survival (24). To evaluate the efficacy of PT in the treatment of psychosomatic symptoms, this systematic review focuses on the psychosomatic symptoms of PT in breast cancer survivors, summarizing and evaluating the evidence from all available randomized controlled trials (RCTs) to obtain relatively robust clinical evidence.

## 2 Methods

### 2.1 Search Strategy

Seven databases (MEDLINE, EMBASE, Cochrane CENTRAL, China National Knowledge Infrastructure, Wangfang, VIP, and China Biology Medicine disc databases) were systematically searched from the database inception through May 18, 2021. The literature lists of relevant review articles and full-text review papers were also cross-checked by different reviewers. The search strategy involved four parts: clinical situation (breast cancer and AIs), intervention (physical therapy), outcomes (psychosomatic symptoms), and study type (randomized controlled trial). The complete search strategies for all the databases can be found in **Supplement 1**. Moreover, the reference review articles, conference summary, and comments on supplementary citations were scrutinized. All the studies included were limited to humans, and there was no language restriction.

### 2.2 Inclusion and Exclusion Criteria

To prevent bias, the inclusion criteria were prespecified according to population, intervention, comparison, and outcome (PICO terms): (P) Types of participants: Participants had a diagnosis of stage I to III ER-positive, or PR-positive breast cancer in accordance with diagnostic criteria (25) and were receiving adjuvant therapy for AIs; (I) Types of interventions: All types of management interventions for psychosomatic symptoms were considered; acupuncture of all types, doses, and courses, and all exercise therapy, which had to meet the definition of “physical activity that is planned, structured and repetitive and has a final or intermediate objective of the improvement or maintenance of physical fitness,” (26) such as tai chi, yoga, aqua aerobics, and resistance exercise. These exercise programs had aerobic/endurance, stretching/flexibility, resistance/strengthening, or combined training as a key component and resulted in significant physiological changes; (C) Types of studies: All RCTs or quasi-experimental studies which examined the effectiveness of all kinds of PTs on AIMSS or psychosomatic symptoms in AIs treated patients with breast cancer; (O) Types of outcome measures:

#### 2.2.1 Primary Outcomes

Pain (subgroup scores of AIMSS including three types of symptoms: pain, stiffness, and grip strength). The score of pain should be measured using scales including the BPI scale, Western Ontario and McMaster Universities Index (WOMAC) scale, VAS scale, and electronic algometer.

Emotional states (including anxiety, depression, sleep disturbance, or fatigue). Emotional state should be measured by standard measurements such as Pittsburgh Sleep Quality Index (PSQI), Hospital Anxiety and Depression Scale (HADS-A), and Functional Assessment of Chronic Illness Therapy-Fatigue (FACIT-Fatigue).

#### 2.2.2 Secondary Outcomes

The QOL score should be measured by self-reported measurements, including the Functional Assessment of Cancer Therapy-General (FACT-G) scale and 36-Item Short Form Survey (SF-36) scale.

The exclusion criteria were prespecified as follows: (1) Not RCT studies such as small case series, protocols, and reviews; (2) Combined use of drugs; (3) No control group or one arm; (4) Duplicate publication; (5) Women with advanced/metastatic breast cancer; and (6) Animal and *in vitro* studies.

### 2.3 Data Extraction and Risk of Bias Assessment

All data were extracted independently by two reviewers, and discrepancies were discussed with the third reviewer. Predesigned forms included study features (name of the first author, year of publication, sample size, and median age), clinical characteristics (participants, interventions, treatment groups, and outcome measures), treatment details, methodological characteristics, and significant results. Two evaluators independently assessed the quality of included studies according to the Cochrane risk-of-bias tool for randomized trials (RoB2). Disagreements were resolved through discussion with another reviewer until consensus was reached. Each study was assigned a low, high, or some concerns risk of bias for six specific areas (the randomization process, deviations from the intended interventions, missing outcome data, measurement of the outcome, and selection of the reported results, and other bias), using information extracted from the papers and supplementary materials and contacting the study authors when needed. The overall evidence and certainty of evidence were evaluated with the Grading of Recommendations Assessment, Development, and Evaluation approach.

### 2.4 Statistical Analyses

Studies with needed data were included in meta-analysis using random- or fixed-effects model according to their effect size to calculate risk ratio and 95% CI. The heterogeneity between trials was determined by *χ* ^2^ test and reported as *I* ^2^. Statistical analyses were performed within Cochrane Program Review Manager Version 5.3 (Cochrane Collaboration, Oxford, UK). Two-sided *P* < 0.05 was considered statistically significant. Studies were grouped according to the type of intervention and the comparator (sham treatment). For studies with multiple control groups, such as real acupuncture *versus* sham acupuncture *versus* wait-list control, the results were divided into pairwise comparisons according to different comparators. When different results of the same study were reported in different publications, the data were merged. Subgroup sensitivity analyses were conducted to explore potential sources of heterogeneity.

## 3 Results

### 3.1 Search Results

**Figure 1:**
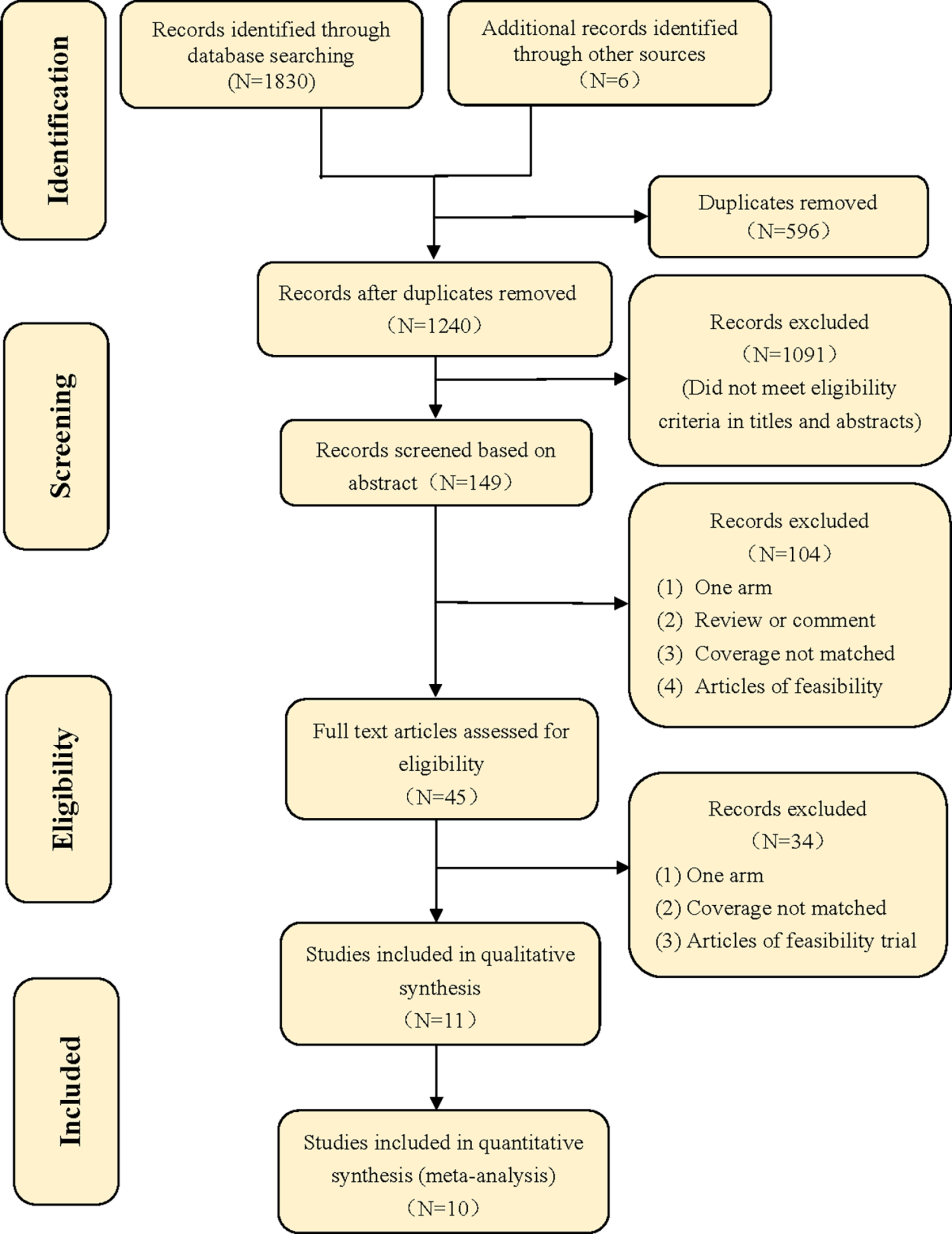
Flow diagram.

A total of 1,836 articles were identified by searching the seven databases, from which six articles (0.3%) came from other sources, such as bibliography and citation searching. After 596 duplicate publications (32%) had been removed, 1,240 records (68%) went through title and abstract screening, after which 1,229 articles (67%) were excluded because they did not meet the inclusion criteria. Eleven studies were included in the systematic review or qualitative synthesis (27–39). One study (9%) (38) was excluded from the meta-analysis as it had incomplete data, and we failed to contact the authors for the missing data. Quantitative synthesis was performed with 10 trials (91%). More details of the process can be found in the study flow diagram (**Figure 1**) (40).

### 3.2 Study Characteristics

#### 3.2.1 Basic Characteristics

Among the 11 clinical trials included, five (45%) were sham controlled (27–30, 37) and six (54%) were open label-controlled trials (31–36). Nine studies (81%) applied a two-group parallel control design, and two studies applied a three-group parallel control design. Five studies (45%) evaluated the efficacy of manual acupuncture and electroacupuncture (27–30, 37), and six (54%) compared different exercise therapies with usual care (31–36). Seven studies (63%) were conducted in United States (27, 29, 30, 33, 34, 36, 37), two (18%) in Australia (32, 38), and one (9%) in Brazil and United Kingdom (32, 35). The study characteristics are shown in **Table 1**.

**Table 1:** Characteristics of trials included in the analysis.

Source^*^	Intervention	Trial design	Sample size (I/C)^a^, dropout (I/C)^a^	Age (I/C)^a^ (year)	Arms (course)	Participant characteristics	Race	Outcome measurement tool	Primary outcome result
Crew et al. (37)	MA	Sham-controlled	43 (23/20), 5 (3/2)	58 (44–77)/57 (37–77)	TA/SA (30 min twice weekly, 6 wk)	Postmenopausal Stage I–III breast cancer Estrogen receptor-positive, progesterone receptor-positive, or both AI for 3 months Pain score on the BPI-SF of ≥3 points	White (39%), Hispanic (55%), Black (3%), Asian (3%)	BPI-SF WOMAC M-SACRAH FACT-G	Positive
**(US)**		2-arm							
Oh et al. (38)	EA	Sham-controlled	32 (16/16), 3 (2/1)	<45 14 (93%) and ≥45 1 (7%)/<45 12 (86%) and ≥45 2 (14%)	TA/SA (30 min twice weekly, 6 wk)	Postmenopausal Stage I–IIIa breast cancer Estrogen receptor-positive, progesterone receptor-positive, or both AI for 6 months Pain score on the BPI-SF of points	Caucasian (86%), Others (14%)	BPI-SF WOMAC FACT-G Blood analysis: CRP, ESR	Positive
**(Australia)**		2-arm							
Mao et al. (27)	EA	Sham-controlled	67 (22/22/23), 8 (3/3/2)	57.5 ± 10.1/60.9 ± 6.5/60.6 ± 8.2	TA/SA/WLC (twice a week for 2 wk; then weekly for 6 wk)	Stage I–III breast cancer AI for 3 months Joint pain ≥3 months Numerical rating scale ≥4 points At least15 days with pain in the preceding 30 days	White (72%), Others (28%)	BPI WOMAC DASH PPT Fatigue: BFI Sleep: PSQI Anxiety: HADS Depression: HADS-Depression	Positive^c^
**(US)** ^b^		3-arm							
Bao et al. (29)	MA	Sham-controlled	51 (25/26), 4 (2/2)	61 (44–82)/61(45–85)	TA/SA (8 weekly for 8 wk)	Postmenopausal Stage I–III breast cancer AI for 1 month Baseline HAQ-DI score ≥3 and/or pain using a 100-point VAS ≥20 Have not received acupuncture treatment in the past 12 months	Caucasian (72%), African American (19%), Other (9%)	HAQ-DI VAS β-endorphin; estradiol; Proinflammatory cytokines: IL-1, -6, -8, -10, -12, -17, IFN-γ, TNF-α Menopausal symptoms: NSABP Hot Flash: HFRDI Sleep: PSQI Depression: CESD Anxiety:HADS-A, Quality of life: Euro Qol	Negative
**(US)** ^b^		2-arm							
Hershman et al. (30)	MA	Sham-controlled	226 (110/59/57), 20 (9/5/6)	60.8 (34.1–80.6)/57.0 (40.6–77.5)/60.6 (27.1–76.0)	TA/SA/WLC (twice a week for 6 wk; then weekly for 6 wk)	Postmenopausal or premenopausal with gonadotropin-releasing hormone agonist Stages I–III breast cancer Estrogen receptor-positive, progesterone receptor-positive, or both AI ≥ 1 month to continue for at least 1 additional year Zubrod performance 0–1 Pain score on the BPI-SF of ≥3 points	White (85%), Black (4.4%), Asian (6.6%), Pacific Islander (0.4%), American Indian (0.4%), others (3.2%)	BPI WOMAC PROMIS PI-SF M-SACRAH FACT-ES	Positive^c^
**(US)**		3-arm							
Baker et al. (31)	EX (Low-frequency, low-magnitude vibrative)	Open label-controlled	31 (14/17), 0	61.6 ± 9.2/61.6 ± 7.8	EX/WLC (20 min of vibration, 3 weekly for 12 wk)	Breast cancer (stage unknown) Taking any bone-altering medications or supplements Able to stand unassisted for sustained periods of time (i.e., 20 min)	Australian (100%)	WOMAC FACT–Fatigue subscale Bone Resorption and Formation: NTx/Cr, P1NP Body Composition and Bone Mineral Density Physical Functioning	Negative
**(Australia)**		2-arm							
Paulo et al. (32)	EX (resistance training followed by aerobic training)	Open label-controlled	36 (18/18), 7 (3/4)	63.2 ± 7.1/66.6 ± 9.6	EX/CG (3 weekly for 36 months)	Aged between 50 and 80 years Stage I–III breast cancer AI for breast cancer No muscle and bone damage	Brazilians (100%)	SF36 EORTC QLQ-C30 EORTC QLQ-BR23	Positive
**(Brazil)**		2-arm							
Baglia et al. (33)	EX (strength-training and aerobic exercise)	Open label-controlled	121 (61/60), 38 (16/22)	62 ± 7/60.5 ± 7	EX/CG (2 weekly for 12 months)	Postmenopausal women, HR-positive, stage I–III BC diagnosed 0.5–4 years prior to enrolment AI for 6 months Arthralgias for ≥2 months, with BPI-SF score≥3/10 4.Pre-existing joint pain allowed if worsened after AI Physically inactive: baseline <90 min exercise/week, no strength training	Non-Hispanic White (85%)	FACT FACT-G FACT-B SF-36 FACIT-Fatigue	Positive
							Hispanic (3%)		
							African American (9%)		
							Asian/Pacific Islander (2%)		
							American Indian (1%)		
**(US)**		2-arm							
Nyrop et al. (34)	EX (Walk)	Open label-controlled	62 (31/31), 9 (7/2)	63.3 ± 6.9/64.4 ± 9.7	EX/WLC (150 min weekly for 6 wk)	Age >21 years Stage 0–III breast cancer AI for 4 weeks Pain score on the BPI-SF of ≥3 points Exercising ≤150 min per week	Caucasian (74%), Others (26%)	VAS WOMAC FACT‐G RAI ASE OEE SEPA	Positive
**(US)**		2-arm							
Fields et al. (35)	EX (Nordic walking)	Open label-controlled	40 (20/20), 0	60 ± 8/66 ± 7	EX/CG (once a week for 6 wk, then four times a week for 6 wk)	Breast cancer (stage unknown) AI for breast cancer Reporting joint symptoms over preceding 12 months	Caucasian (100%)	BPI-SF PSEQ CES-D SF-36	Positive
**(UK)**		2-arm							
Irwin et al. (36)	EX (strength-training and aerobic exercise)	Open label-controlled	121 (61/60), 38 (16/22)	62 ± 7/60.5 ± 7	EX/CG (2 weekly for 12 months)	Postmenopausal women, HR-positive, stage I–III BC diagnosed 0.5–4 years prior to enrolment AI for 6 months Arthralgias for ≥2 months, with BPI-SF score≥3/10 Pre-existing joint pain allowed if worsened after AI Physically inactive: baseline <90 min exercise/week, no strength training	Non-Hispanic White (85%)	BPI WOMAC DASH Grip strength	Positive
							Hispanic (3%)		
							African American (9%)		
							Asian/Pacific Islander (2%)		
							American Indian (1%)		
**(US)**		2-arm							

*Sources of funding for the included studies are provided in the **Supplement 1**.

a I/C, data of Intervention group/data of control group(s);

b Different outcomes were reported in separate publications; the data were merged.

c Primary results of comparisons (experimental intervention vs. sham control(s) and experimental intervention vs. waitlist control) were both positive.

NM, no mention; EA, electroacupuncture; MA, manual acupuncture; AI, aromatase inhibitor; TA, true acupuncture group; SA, sham acupuncture group; WLC, waitlist control; EX, exercise; CG, control group; BPI-SF, Brief Pain Inventory–Short Form; WOMAC, Western Ontario and McMaster Universities Osteoarthritis Index; M-SACRAH, Modified Score for the Assessment and Quantification of Chronic Rheumatoid Affections of the Hands; FACT-G, Functional Assessment of Cancer Therapy–General; BFI, Brief Fatigue Inventory; PSQI, Pittsburgh Sleep Quality Index; HADS, Hospital Anxiety and Depression Scale; HAD-DI, The Health Assessment Questionnaire—the disability index; NSABP, National Surgical Adjuvant Breast and Bowel Project; HADS-A, Hospital Anxiety and Depression Scale—the anxiety subscale; PROMIS PI-SF, The PROMIS Pain Impact-Short Form; CES-D, Center for Epidemiological Studies Depression; PSEQ, Pain Self-Efficacy Questionnaire; DASH, The Disabilities of the Arm, Shoulder, and Hand questionnaire; PPT, The Physical Performance Test; BMI, body mass index; %FM, percent body fat; LBM, lean body mass; BMD, bone mineral density; FSI, Fatigue Symptom Inventory; AUSCAN, Australian/Canadian Hand Osteoarthritis Index; HFRDIS, Hot Flash Related Daily Interference Scale; EORTC QLQ-C30, European Organization for Research and Treatment of Cancer questionnaire entitled “Quality of life Questionnaire version 3.0; EORTC QLQ-BR23, European Organization for Research and Treatment of breast cancer module; FACT-ES, Functional Assessment of Cancer Therapy—the endocrine subscale; FACT-B, Functional Assessment of Cancer Therapy–Breast cancer; FACIT-Fatigue, Functional Assessment of Chronic illness Therapy–Fatigue; SF-36, the MOS item short from health survey; VAS, visual analog scale; RAI, rheumatology attitudes index; ASE, arthritis self‐efficacy scale; OEE, outcome expectations from exercise; SEPA, self-efficacy for physical activity.

#### 3.2.2 Population

A total of 830 patients were enrolled in the 11 studies, and 10 studies with 795 patients were included in the meta-analysis. The sample sizes included in the studies ranged from 29 to 226, with 387 participants (49%) in the acupuncture trials (27–30, 37–39) and 411 (51%) in the exercise trials (31–36). Eleven studies reported the mean ages of participants, ranging from 57 to 66 years (27, 29–37), and one study reported age ranges (38). At the time of enrolment, all participants were using AIs, including anastrozole, letrozole, exemestane, etc. Nine studies (81%) reported the patients were diagnosed with breast cancer (staged as I–III), and six studies (54%) (29, 30, 33, 36–38) reported the patients were postmenopausal. The dropout rate of four studies (36%) was zero (31, 34, 35, 38). Some studies reported that their inclusion criteria were women who experienced any joint symptoms while taking AIs, and eight studies (72%) set the minimum pain score that met the inclusion criteria (27, 29, 30, 33, 34, 36–38).

#### 3.2.3 Interventions

The interventions included acupuncture [manual acupuncture (29, 30, 37, 39) and electroacupuncture (27, 38)], exercise (magnitude vibrative (31), walking (34, 35), and training [32, 33, 36)], sham acupuncture, and no treatment. Four studies (36%) used acupuncture therapy twice a week for 30 min for 2 to 6 weeks (27, 30, 37, 38), and one study (9%) used acupuncture therapy eight times a week for 8 weeks (29). Two studies (18%) surveyed walking plans, one of which was Nordic walking, which utilizes walking with hand-held poles (35). Another walking study was based on a family exercise program, walking 150 min a week (34). Three studies (27%) used a combination of resistance training plus aerobic exercise (32, 33, 36). The intervention time of each study is different, ranging from 6 weeks to 12 months. There were different reports on the intensity of exercise intervention. One study (9%) reported the ideal exercise intensity level, with a target of 60 to 80% of the maximum heart rate, based on the VO_2_ maximum test (36). The majority of studies included at least 150 to 200 min of exercise per week.

#### 3.2.4 Outcomes

Pain was one of the most important components of psychosomatic symptoms. In five studies (45%), the BPI scale was used to assess the worst pain, worst stiffness, and pain severity associated with AIMSS in patients diagnosed with breast cancer (28, 30, 35–37). In five studies (45%), the WOMAC was used to assess the severity of knees or hips osteoarthritis (28, 31, 34, 36, 37). The global score of the PSQI was used to measure sleep condition in two studies (18%) (27, 29). Anxiety was assessed with the anxiety subscale of the HADS-A in two studies (18%) (27, 29). FACIT-Fatigue is a 13-item subscale to assess fatigue-related concerns in two studies (18%) (31, 33). QOL was measured by the FACT-G questionnaires in two studies (18%) (33, 34) and SF-36 in three studies (27%) (32, 33, 35).

### 3.3 Methodological Quality of Clinical Studies

**Figure 2:**
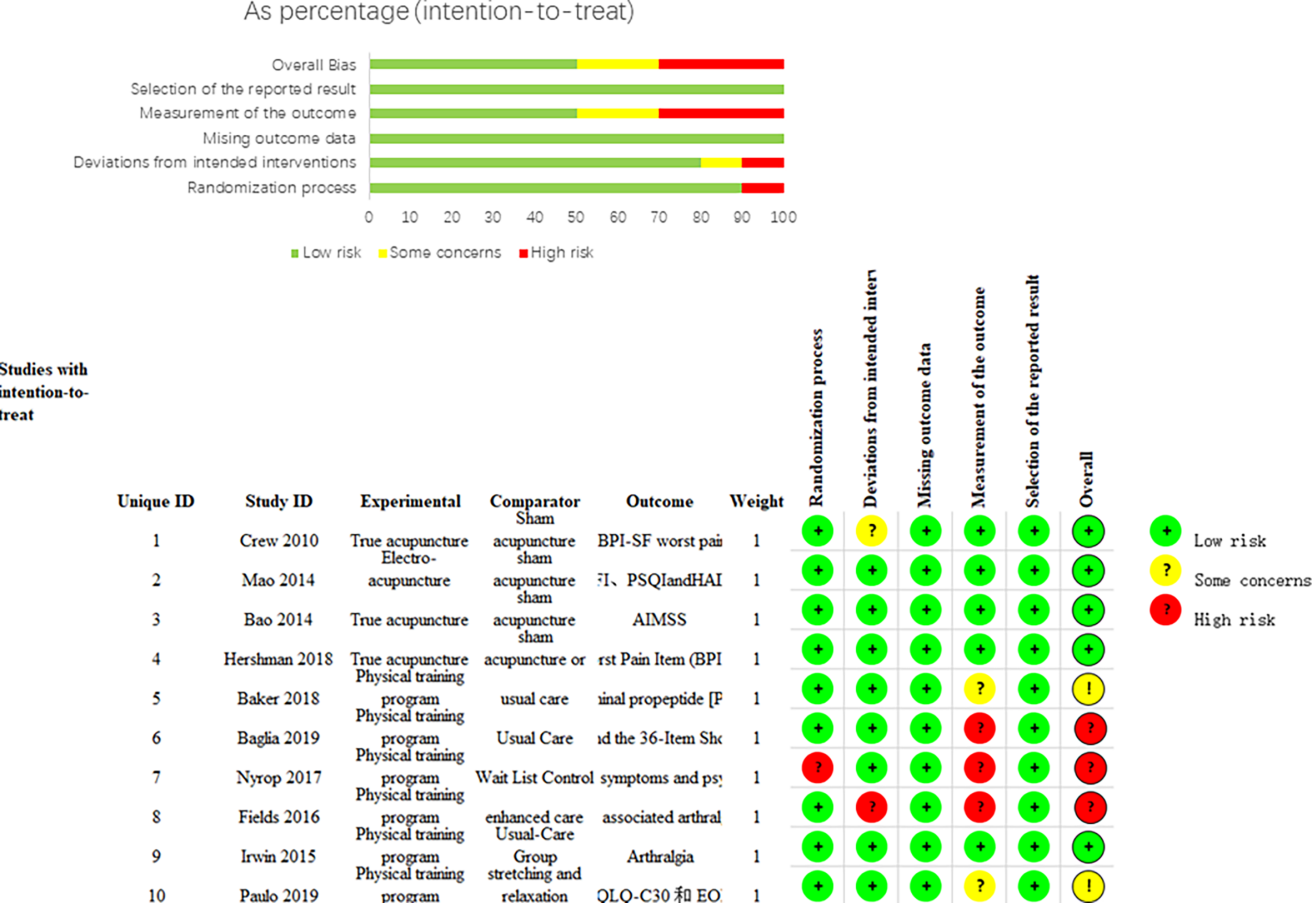
Each “Risk of bias” item for each included study.

Details of the risk assessment of bias in the included studies are documented in **Figure 2**. Four studies (36%) (27, 29, 30, 36, 37) were of high quality. Because of the nature of the exercise intervention, it was not practical to ensure blindness of participants and outcome assessors. However, the measurements of psychosomatic symptoms were always the result of the patients’ reported outcomes. Therefore, detection bias was objectively presented when participants and outcome assessors could not be blinded to the intervention. Thus, the five open-label studies (45%) (31–35) without sham exercises were rated as having a high or some concerns risk of bias for blinding of the participants and outcome assessors. One study (9%) (34) reported randomization errors, and although the cause and time of the error were not reported, we judged it to be a high-risk selection bias (random and allocation concealment). One study did not use appropriate analysis to estimate the effect of assignment to intervention, and we judged (9%) (35) it to be a high-risk selection bias (deviations from the intended interventions). Details of this study are presented in the **Supplement 1**.

### 3.4 Outcomes of Acupuncture and Exercise

#### 3.4.1 Interventions of Pain

Seven studies used outcomes reported by participants to assess pain symptoms (28, 30, 37), which included the BPI scale and WOMAC. The BPI scale is composed of three subscales: pain-related interference, pain severity, and worst pain. In acupuncture trials (28, 30, 37), three subscales were analyzed, but in exercise trials (35, 36), only analyzed one subscale of worst pain. WOMAC is composed of four subscales: the pain, stiffness, function (difficulty), and normalized subscales. In the meta-analysis of acupuncture and exercise trials, we only included the pain subscale of WOMAC to assess the effect of interventions.

**Figure 3:**
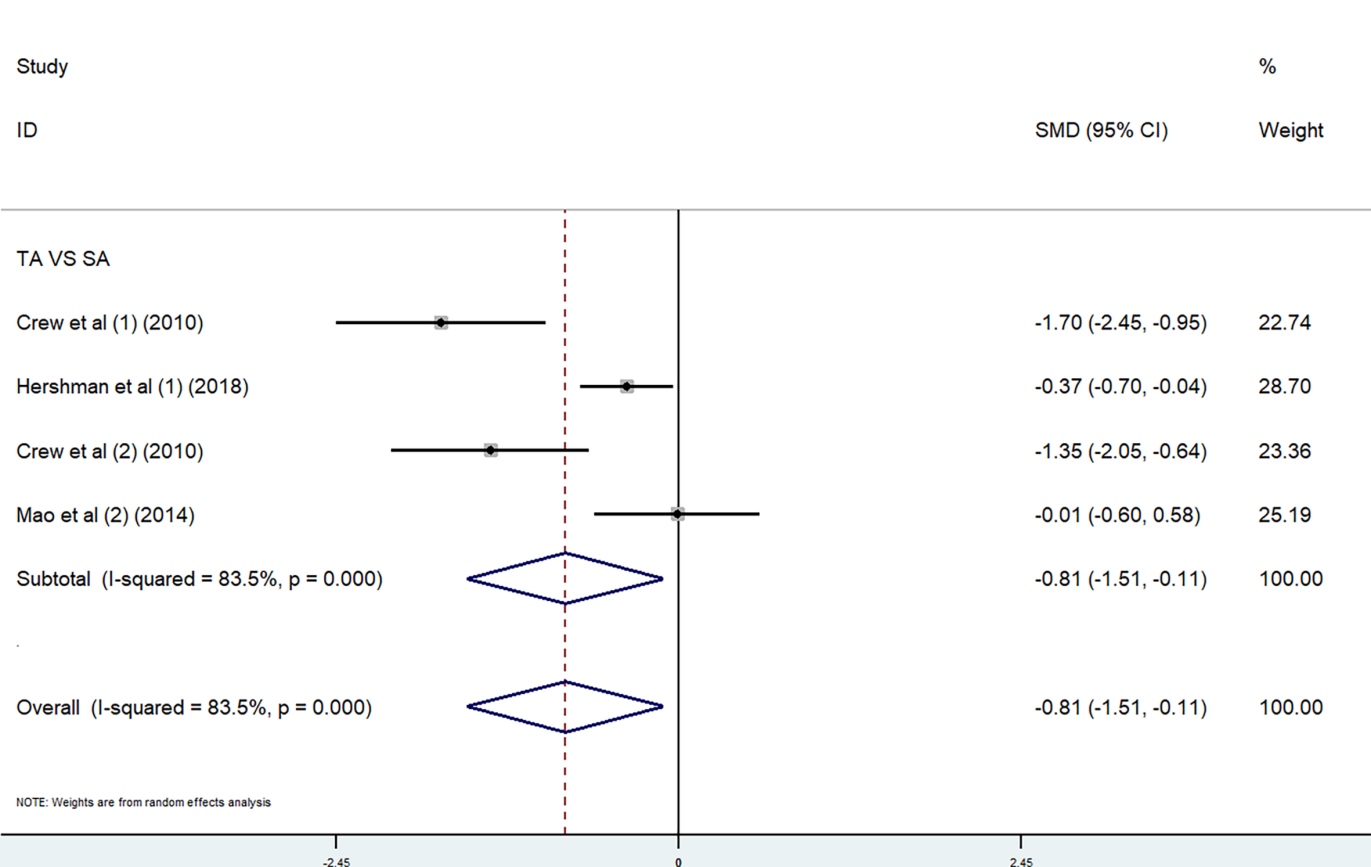
Overall change of acupuncture trials in pain, combined using (1) BPI worst pain subscale and (2) WOMAC pain subscale.

*Acupuncture.* We performed a meta-analysis on the effect of acupuncture on worst pain. Because of the different scoring systems, SMD was used. In the meta-analysis, two studies (30, 37) used the BPI worst pain scores and two studies (28, 37) used the WOMAC pain subscale. Given the high heterogeneity of the four studies (p < 0.00001, *I* ^2^ = 83.5%), we used a random-effects model in the combined effects analysis. The true-acupuncture (c) group was better than the sham-acupuncture (SA) group [SMD = −0.81, 95% CI (−1.51, −0.11)] (**Figure 3**). The significant heterogeneity might result from the diversity of outcome assessment tools and acupuncture interventions used in the studies.

**Figure 4:**
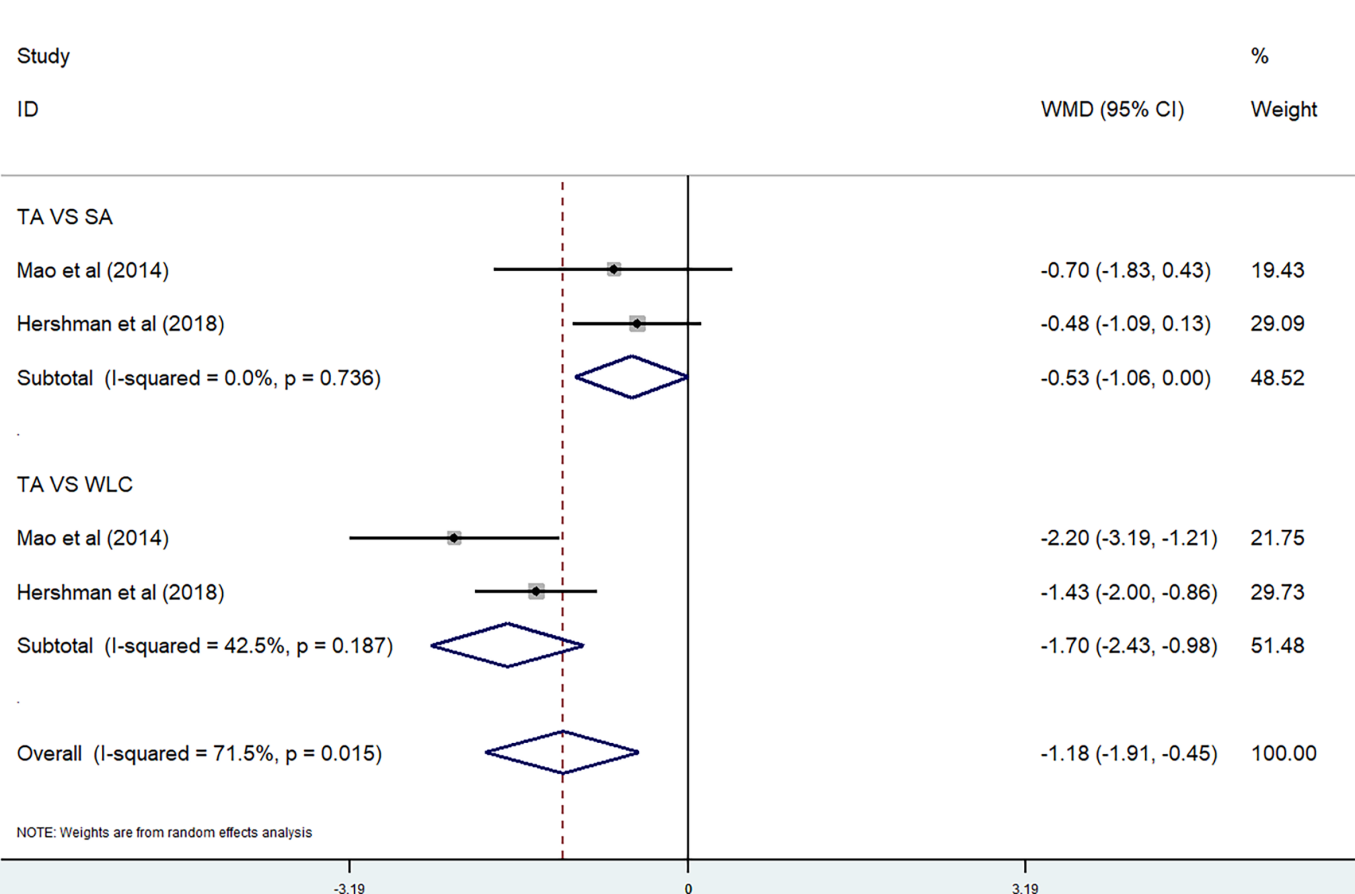
BPI Pain Severity score in acupuncture trials.

**Figure 5:**
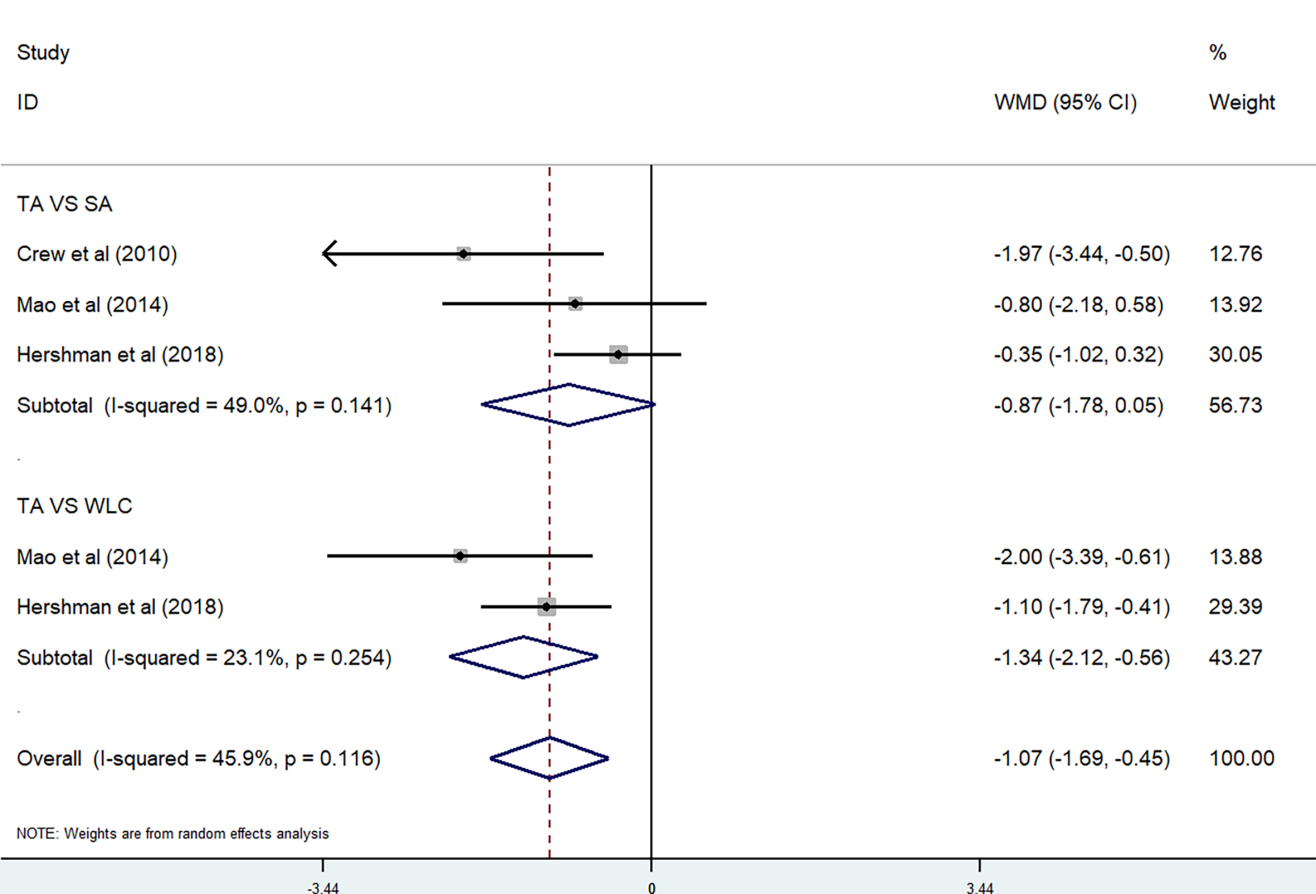
BPI Pain-Related Interference score in acupuncture trials.

Two studies were included in the meta-analysis on BPI Pain Severity score (28, 30). Because of the high heterogeneity (P=0.015 < 0.05, *I* ^2^ = 71.5%), we divided them into two subgroups according to the control method used. No significant differences were observed between the TA group and SA group (P = 0.736, *I* ^2^ = 0%) [WMD = −0.53, 95% CI (−1.06, 0)], while there were also no significant differences between the TA group and waitlist control (WLC) group (P = 0.187, *I* ^2^ = 42.5%) [WMD = −1.70, 95% CI (−2.43, −0.98)] (**Figure 4**). Three studies were included in the meta-analysis on BPI Pain-Related Interference score (28, 30, 37). Because of the high heterogeneity (P=0.116, *I* ^2^ = 45.9%), we divided them into two subgroups based on the control method used. There were no significant differences between the TA group and SA group (P = 0.141, *I* ^2^ = 49.0%) [WMD = −0.87, 95% CI (−1.78, 0.06)], while there were also no significant differences between the TA group and waitlist control (WLC) group (P = 0.254, *I* ^2^ = 23.1%) [WMD = −1.34, 95% CI (−2.12, −0.56)] (**Figure 5**).

**Figure 6:**
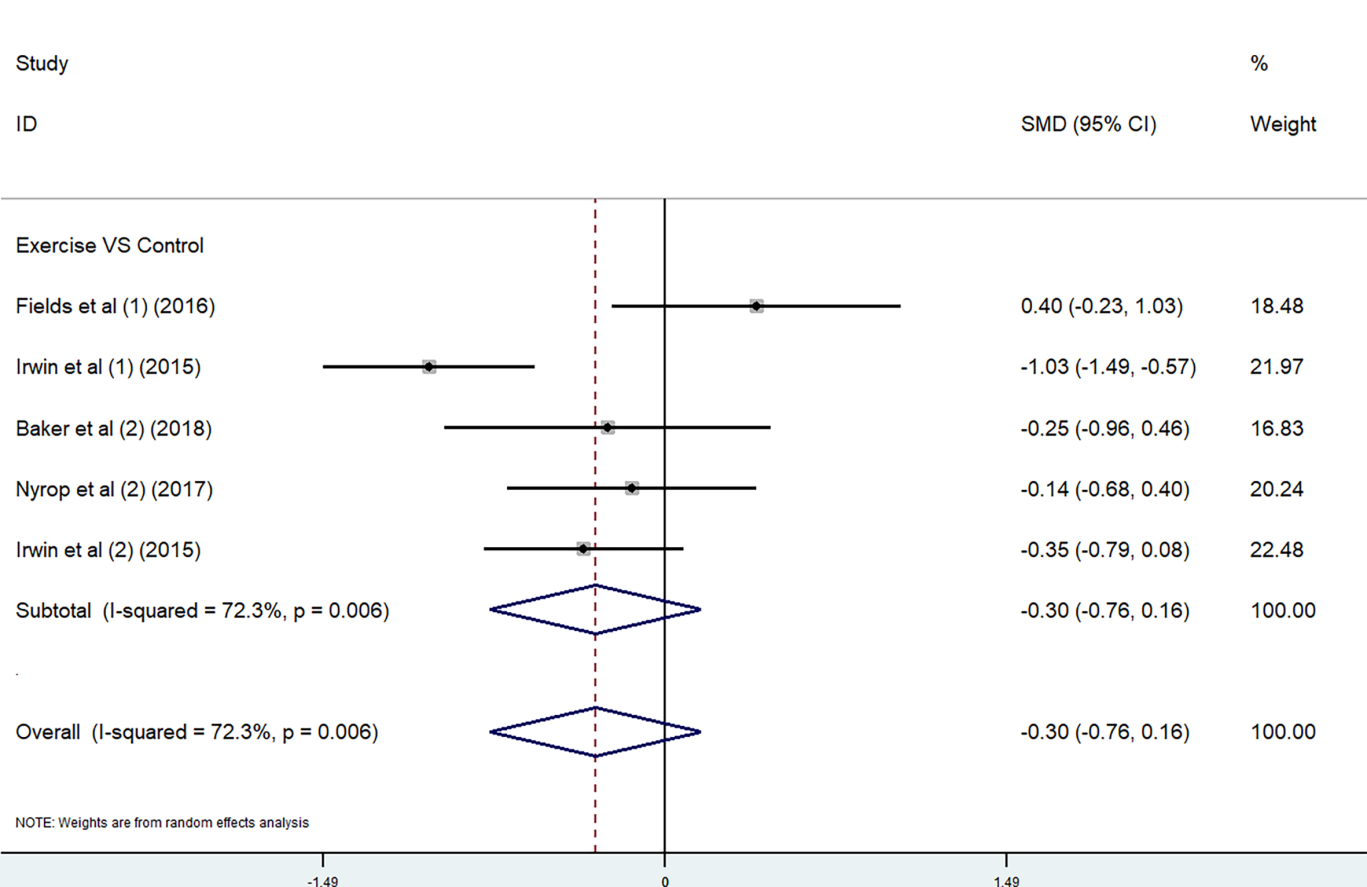
Overall change of exercise trials in pain, combined using (1) BPI worst pain subscale and (2) WOMAC pain subscale.

*Exercise.* The effect of exercise on pain was performed by using BPI worst pain scores and WOMAC pain subscale. Because of the different scoring systems, SMD was used. In the meta-analysis, two studies (35, 36) used the BPI worst pain scores and three studies (31, 34, 36) used the WOMAC pain subscale. Considering the high heterogeneity of these five studies (P =0.006, *I* ^2^ = 72.3%), we adopted a random-effects model in the comprehensive effects analysis. There were no significant differences between the exercise group and the control group (P =0.006, *I* ^2^ = 72.3%) [SMD = −0.30, 95% CI (−0.76, 0.16)] (**Figure 6**). The considerable statistical heterogeneity of studies in the meta-analysis might result from the range of exercise interventions utilized between the studies and the wide range of outcome assessment tools used. Due to the lack of available data, the other subscales were not analyzed in exercise trials.

#### 3.4.2 Interventions of Anxiety, Sleep Disturbance, and Fatigue

**Figure 7:**
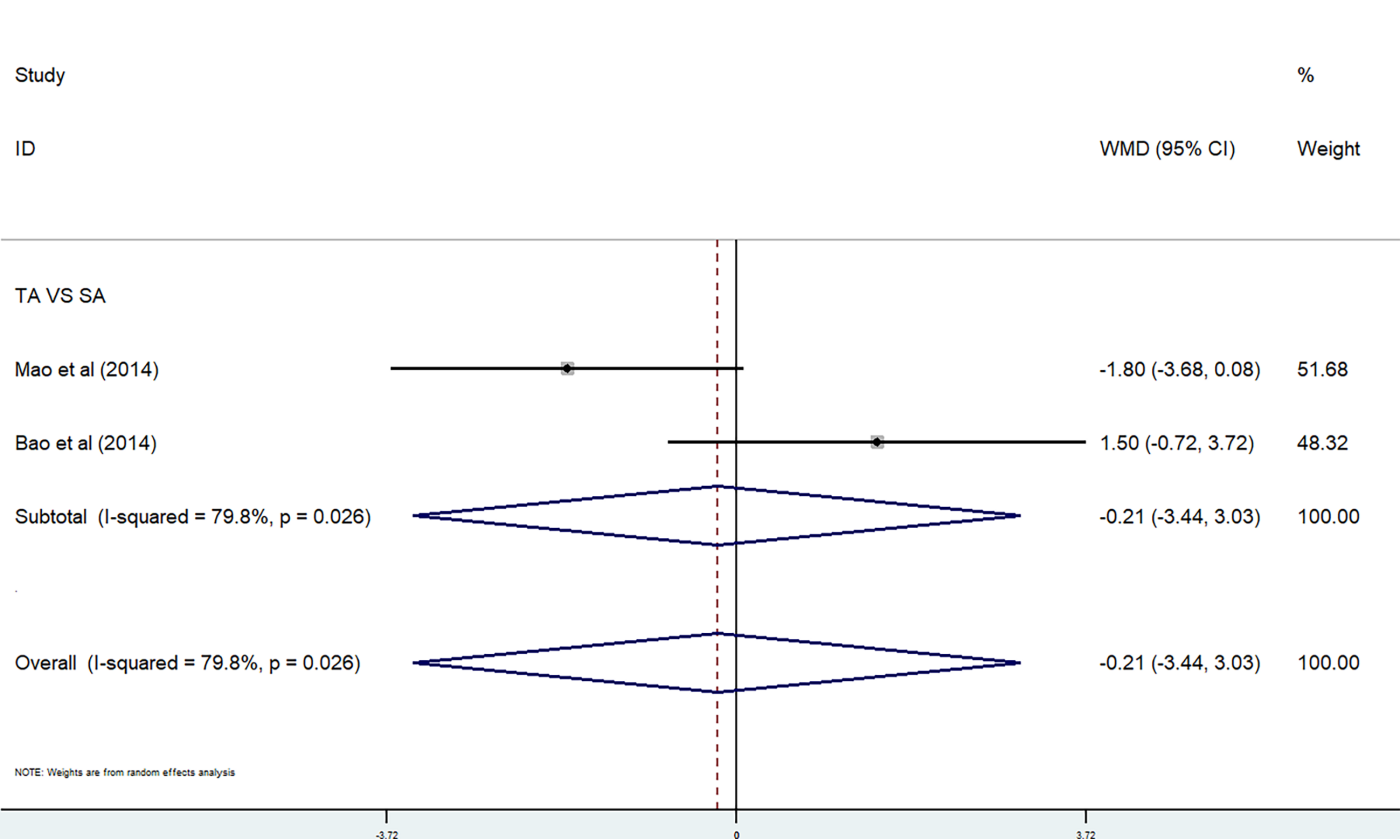
HADS-A subscale in acupuncture trials.

**Figure 8:**
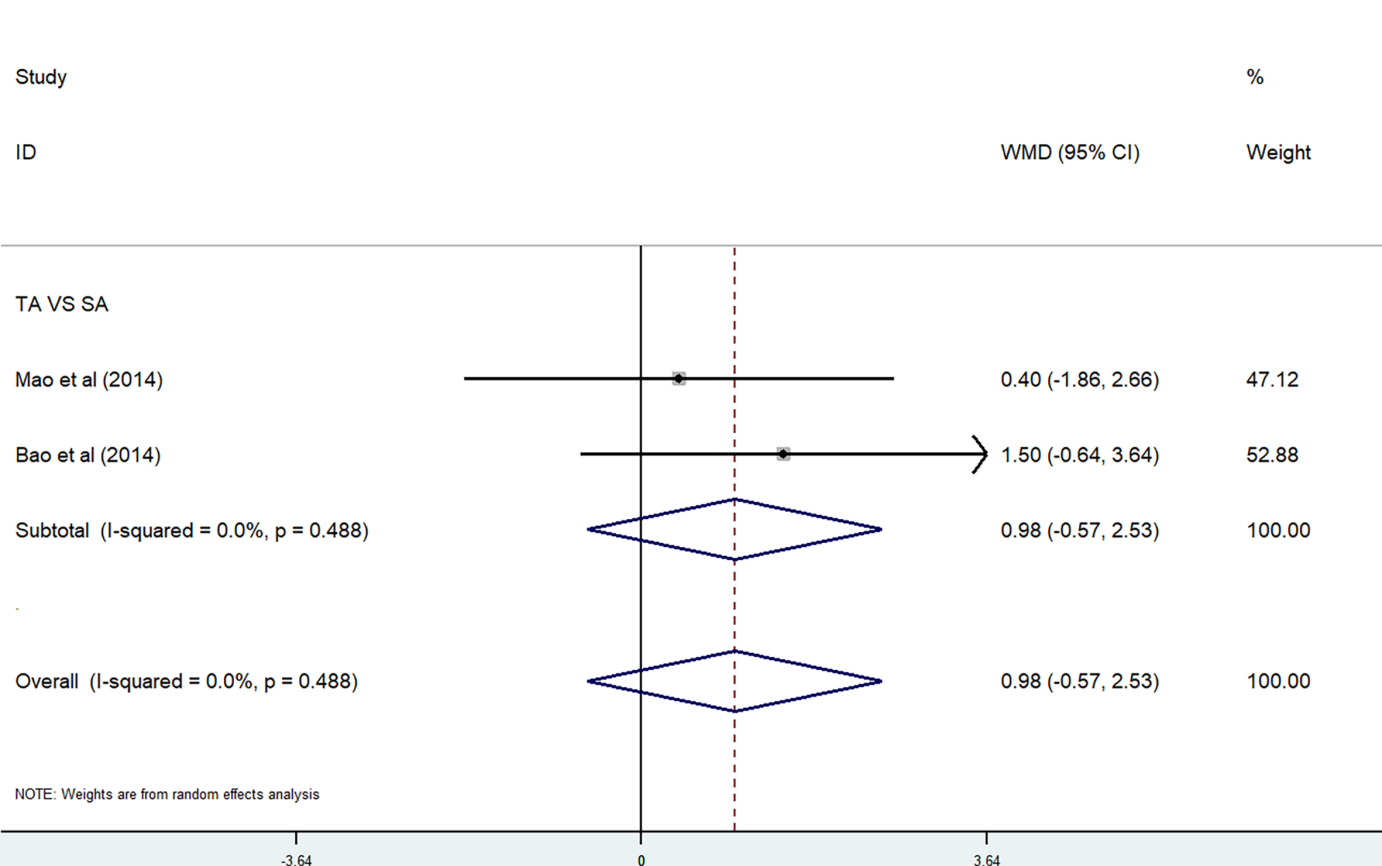
PSQI subscale in acupuncture trials.

*Acupuncture.* The effect of acupuncture on anxiety and sleep disturbance was performed in a meta-analysis by using HADS-A and PSQI. In the meta-analysis, two studies used the HADS-A subscale (27, 29). Because of the high heterogeneity, we used a random-effects model. There were no significant differences between the TA group and the SA group (P = 0.026<0.05, *I* ^2^ = 79.8%) [WMD = −0.21, 95% CI (−3.44, 3.03)] (**Figure 7**). Two studies were included in the meta-analysis on PSQI subscale (27, 29). Because of the low heterogeneity, we used a fixed-effects model. There were also no significant differences between the TA group and the SA group (P = 0.488, *I* ^2^ = 0%) [WMD = 0.98, 95% CI (−0.57, 2.53)] (**Figure 8**).

**Figure 9:**
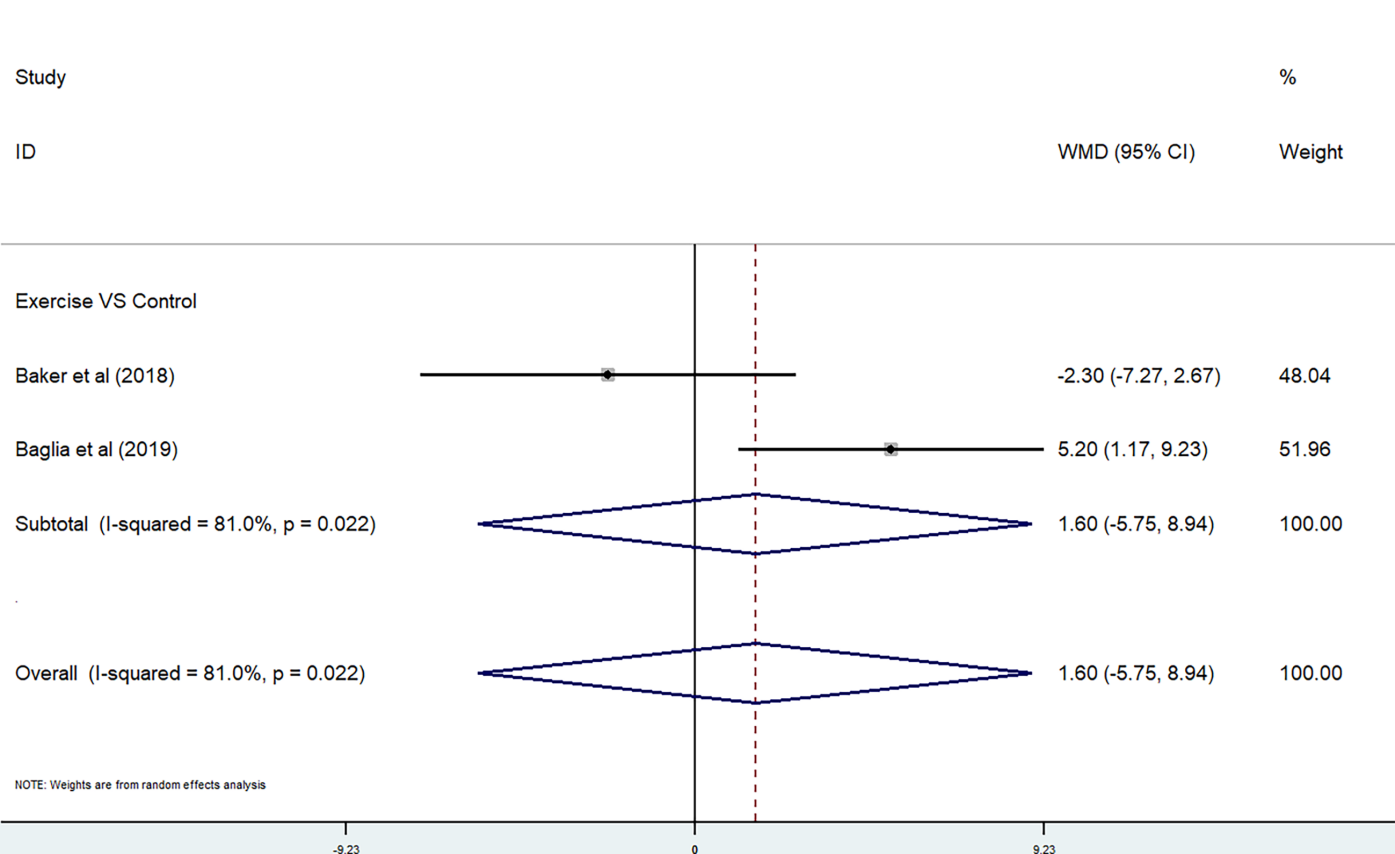
FACIT-Fatigue subscale in exercise trials.

*Exercise.* The effect of exercise on fatigue was performed in a meta-analysis by using FACIT-Fatigue. In the meta-analysis, two studies used the FACIT-Fatigue subscale (31, 33). Because of the high heterogeneity, we used a random-effects model. There were no significant differences between the exercise group and the control group (P = 0.022<0.05, *I* ^2^ = 81.0%) [WMD = 1.6, 95% CI (−5.75, 8.94)] (**Figure 9**).

#### 3.4.3 Interventions of QOL

**Figure 10:**
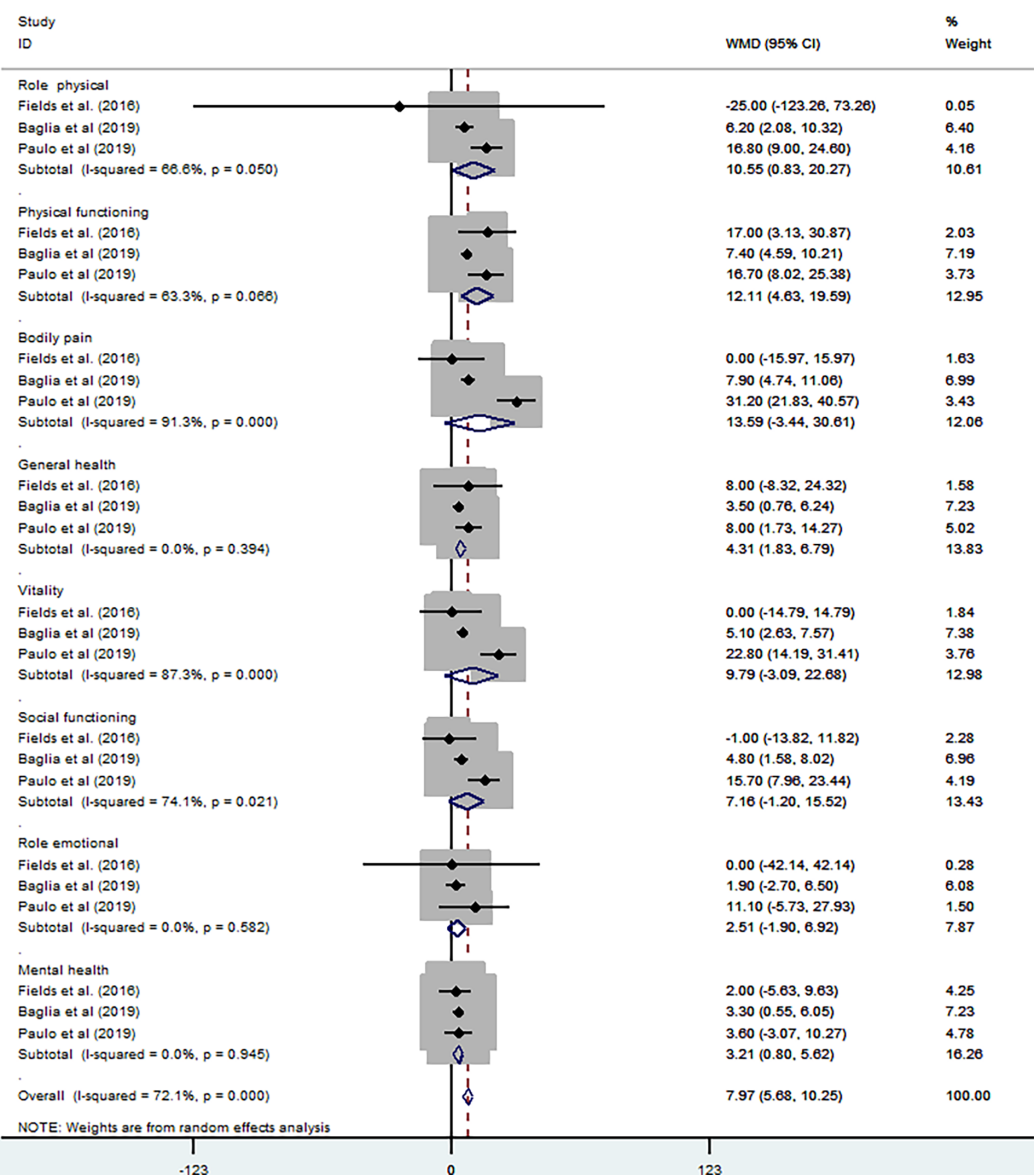
SF-36 subscale in exercise trials.

*Exercise.* Three studies used SF-36 to assess health-related QOL (32, 33, 35). The subscales of SF-36 can be grouped into a Physical Component Score and a Mental Health Component Score. The higher the score, the better the health status. Using a random-effects model, the results are from eight subscales: role physical (P = 0.05, *I* ^2^ = 66.6%) [WMD = 10.55, 95% CI (0.83, 20.27)]; physical functioning (P = 0.066, *I* ^2^ = 63.6%) [WMD = 12.11, 95% CI (4.63, 19.59)]; body pain (P = 0, *I* ^2^ = 91.3%) [WMD = 13.59, 95% CI (−3.44, 30.61)]; general health (P = 0.394, *I* ^2^ = 0%) [WMD = 4.31, 95% CI (1.83, 6.79)]; vitality (P = 0, *I* ^2^ = 87.3%) [WMD =9.79, 95% CI (−3.09, 22.68)]; social functioning (P = 0.021, *I* ^2^ = 74.1%) [WMD = 7.16, 95% CI (−1.20, 15.52)]; emotional role (P = 0.582, *I* ^2^ = 0%) [WMD = 2.51, 95% CI (−1.90, 6.92)], and mental health (P = 0.945, *I* ^2^ = 0%) [WMD = 3.21, 95% CI (0.8, 5.62)]. The overall result of meta-analysis showed the effect of exercise resulted in improvements in health-related QOL (P = 0, *I* ^2^ = 72.1%) [WMD = 7.97, 95% CI (5.68, 10.25)] (**Figure 10**).

**Figure 11:**
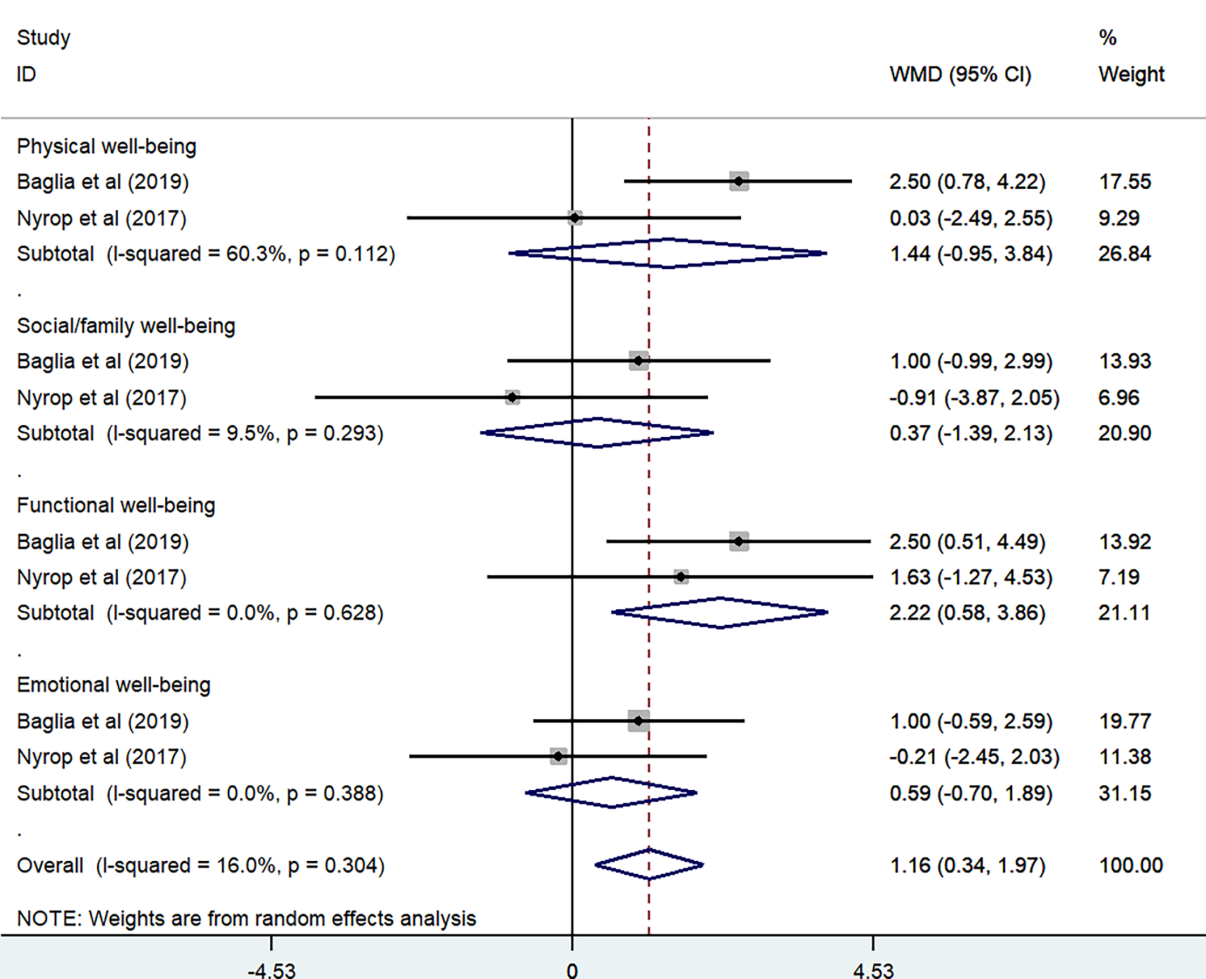
FACT-G subscale in exercise trials.

FACT-G is a 27-item questionnaire assessing physical well-being, social/family well-being, emotional well-being, and functional well-being. Two studies were included in the meta-analysis on FACT-G to assess cancer-specific QOL (33, 34). The results using a random-effects model from the four subscales included physical well-being (P = 0.112, *I* ^2^ = 60.3%) [WMD = 1.44, 95% CI (−0.95, 3.84)]; social/family well-being (P = 0.293, *I* ^2^ = 9.5%) [WMD = 0.37, 95% CI (−1.39, 2.13)]; functional well-being (P = 0.628, *I* ^2^ = 0%) [WMD = 2.22, 95% CI (0.58, 3.86)], and emotional well-being (P = 0.388, *I* ^2^ = 0%) [WMD = 0.59, 95% CI (−0.70, 1.89)]. The overall change of meta-analysis showed the effect of exercise also resulted in improvements in health-related QOL (P = 0.304, *I* ^2^ = 16%) [WMD = 1.16, 95% CI (0.34, 1.97)] (**Figure 11**).

### 3.5 Adverse Effects

Only minor adverse events were reported, which need not medical evaluation or intervention. Three acupuncture studies have reported bruising of the skin and subcutaneous tissues, slight pain from the application of treatment to the skin, or presyncope (27, 30, 38). Three acupuncture studies reported no adverse events or no mention of adverse events (29, 37, 39). Reports of adverse events were seen in two exercise studies, such as pain, syncope, increased swelling, and extreme distress during a training session (31, 35). Four exercise studies recorded no adverse events or did not mention adverse events (32–34, 36).

## 4 Discussion

This was the first systematic review and meta-analysis to evaluate the effect of PTs on psychosomatic symptoms induced by AIs in breast cancer patients. The systematic review included 11 RCTs involving 830 participants with breast cancer, and 10 studies with 798 participants were included in meta-analysis. The results show that acupuncture, compared with no treatment, could significantly improve the worst pain score, pain-related interference score, and pain severity score of the BPI scale. However, compared with no treatment, supervised exercise did not significantly improve the worst pain score of the BPI scale. The results are consistent with previous findings (21). Our results seem to suggest the superiority of acupuncture to exercise in pain improvement, though for now it is not recommended by guidelines (41, 42) and the quality of the evidence was of low level. More direct evidence that compares acupuncture and exercise therapy is needed. In addition to this, exercise was reported to result in a perceived improvement in patients’ QOL, suggesting its potency in enhancing patients’ well-being. The evidence suggests that both acupuncture and exercise result in little to no change in anxiety, sleep disturbance, and fatigue in patients suffering from psychosomatic symptoms, but the quality of the evidence for this outcome was not high, either. Relatively, few adverse events of acupuncture and exercise were reported, which was consistent with previous findings (18, 21).

**Figure 12:**
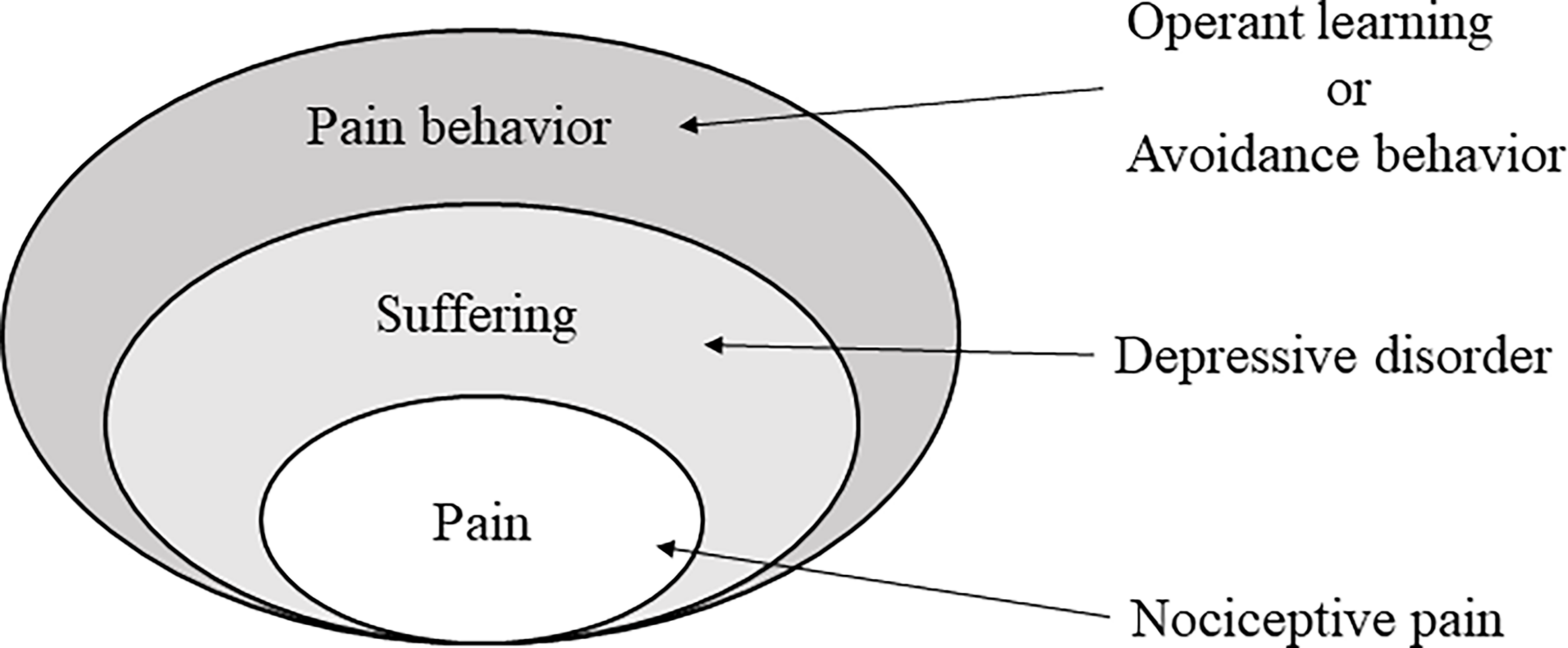
The chronic pain of multiaxial hierarchical structure.

The worldwide use of cytotoxic chemotherapy or other antitumor means is partly encouraged by AIs antitumor efficacy and better safety profile. However, they are not free of adverse effects (43). AI-associated arthralgia (AIA) is characterized by symmetrical joint pain, mostly affecting hands, wrists, and knees, which might have significant impact on patients’ QOL and compliance of treatment (44). On the individual level, breast cancer could induce anxiety and depression, as patients cannot help but worry about their future. Pain might aggravate this process, although very little is known about the pathophysiology of AIA (44). Various researches have shown significant association between perceived stress caused by pain and psychosomatic complaints (45). The ​definition of pain states that it is a subjective sensory and emotional experience. Pain has always been an unpleasant sensation, which has to do with both our psychosomatic conditions and previous experience of pain (46) (**Figure 12**). The notably improved clinical outcomes in breast cancer juxtaposed with significant treatment-related morbidity and mortality has led to interest in the development of de-escalated therapeutic strategies with the goal of maintaining or further improving oncologic outcomes while reducing short- and long‐term toxicity and treatment-related distress (47). Currently explored strategies include replacing, reducing, or omitting cytotoxic chemotherapy; reducing dose or volume of radiotherapy; incorporation of less-invasive surgical approaches; and adjuvant therapies (48). Several clinical trials have provided treatments for AIA, among which are alternative approaches, such as physical exercise, herbal remedies, acupuncture, and yoga, though most evidence are of low quality. Presently, there were no standard, uniformly accepted treatments for AIA, and the majority of the proposed algorithms were based on anecdotal reports or derived from experiences in other pathologies, rather than from specific trials (49). This study applies more quality evidence to prove acupuncture was associated with significant reductions in pain intensity and exercise and might improve QOL in breast cancer patients treated with AIs. Therefore, we tried to apply more stringent inclusion of recent high-quality trials to ensure the quality of RCTs and improve the credibility of evidence. This can serve as a demonstration for future design of de‐escalation studies in the patient population.

Admittedly, some guidelines recommend exercise as a part of the routine lifestyle of women with breast cancer (41, 42). Furthermore, several RCTs evaluate different exercises, such as yoga, tai-chi, swimming, walking, and Pilates, which emphasized the benefits of supervised exercise on AIA, that is, pain, stiffness, grip strength, and QOL improvements (35, 50). Previous studies had shown that exercise had positive impact and significantly improved a wide range of functional, psychological, and physiological markers in individuals regardless of the type of cancer and stages of treatment (51–53). Exercise might be associated with small decreases in pro-inflammatory markers (54). Specifically, TNF and CRP were lower after training, which may have clinical relevance as both are considered as prognostic biomarkers in cancer and produced a more optimal antitumor environment (54). And our results were well in accordance with an earlier study including seven RCTs with a total of 400 enrolled patients, but didn’t provide explicit evidence in favor of the benefits of supervised exercise in AIA (21). Recently, acupuncture for their role in reducing pain has been increasingly appreciated. As sham acupuncture helps avoid bias in assessing the specific outcome of acupuncture, multiple researches and reviews had found the potential efficacy of acupuncture in reducing AIA (15, 18, 55). Available evidence has confirmed acupuncture as a key component of pain management. Furthermore, the present meta-analysis found acupuncture to be associated with demonstrable pain reduction compared to exercise and no treatment, which might show superiority of acupuncture over exercise in pain alleviation. However, few trials evaluated the influence between acupuncture and exercise. Thus, further research is needed to investigate the superiority of acupuncture to exercise in alleviating AIA.

The effect of acupuncture on life quality of patients with breast cancer treated with AIs was investigated in few studies. We only evaluated the effect of exercise on QOL in women with AIMSS. The wide range of symptomatology of AIMSS and the potential severity of symptoms could affect multiple facets of health and well-being for women (56). The results of health-related QOL and cancer-specific QOL, which was assessed using the SF-36 and FACT-G, respectively, showed that exercise training led to a moderate improvement in QOL. Nonetheless, substantial heterogeneity lowered the grade of evidence from high to moderate. Several potential mechanisms might interpret the benefits of exercise in cancer patients. First, exercise helps promote body composition and psychological benefits through maintaining cardiovascular function and metabolic parameters (57). Second, exercise elevates emotional experiences by the neural factors and neurotransmitter systems, such as the endocannabinoid system (58). Third, exercise also enhances immune function and decreases inflammatory factors, which are the possible causes of carcinogenesis (59, 60).

## 5 Limitations

Several limitations were observed and lowered the evidence grade from high to moderate. First, substantial heterogeneity damaged the credibility of the evidence, which prevented us from drawing a high-quality conclusion, although sensitivity analyses were attempted through subgroup analyses. Second, the limited number of trials included for each comparison in the meta-analysis caused unfeasibility of funnel plots, which could not fully evaluate publication bias. Third, both real acupuncture and sham acupuncture can improve pain scores, which means that sham acupuncture may provide a therapeutic benefit. The mechanism may be triggering the release of endorphins or activating pain-related neural matrix (15). Fourth, it is hardly possible to blind the participants in certain studies, such as the treatment arms in the exercise groups. Fifth, several acupuncture trials did not successfully blind their treatment arms. This may have resulted in a bias from positive patient expectations. Finally, baseline analgesic use was not specified. Consequently, variations in analgesic type and dose among participants within each study and between studies are also likely to contribute to heterogeneity.

## 6 Conclusions

Out findings show that based on moderate-level evidence, acupuncture can significantly reduce pain intensity and exercise may improve QOL in breast cancer patients treated with AIs. However, acupuncture or exercise training could not significantly improve some psychosomatic symptoms (such as anxiety, sleep disturbance, and fatigue).

## Data Availability Statement

The original contributions presented in the study are included in the article/**Supplementary Material**. Further inquiries can be directed to the corresponding author.

## Author Contributions

Study conception and design: X-YZ, ZL, CC, and PZ. Acquisition, analysis, and/or interpretation of data: R-LF, B-RC, R-YL, R-TW, LX, YW, and XT. Final approval and overall responsibility for this published work: PZ and ZL. All authors contributed to the article and approved the submitted version.

## Funding

This work was supported by the 2020 Dongzhimen Hospital Affiliated to Beijing University of Chinese Medicine Science and Technology Innovation Special Fund (DZMKJCX-2020-027).

## Conflict of Interest

The authors declare that the research was conducted in the absence of any commercial or financial relationships that could be construed as a potential conflict of interest.

## Publisher’s Note

All claims expressed in this article are solely those of the authors and do not necessarily represent those of their affiliated organizations, or those of the publisher, the editors and the reviewers. Any product that may be evaluated in this article, or claim that may be made by its manufacturer, is not guaranteed or endorsed by the publisher.
